# Disaster-related home loss, mental health, and risk of cognitive disability: causal mediation analysis using longitudinal data of disaster survivors

**DOI:** 10.1093/aje/kwaf208

**Published:** 2025-09-22

**Authors:** Sakurako S Okuzono, Koichiro Shiba, David T Zhu, Sarah Oh, Yu-Tien Hsu, Aki Yazawa, Hiroyuki Hikichi, Jun Aida, Katsunori Kondo, Henning Tiemeier, Ichiro Kawachi

**Affiliations:** Department of Social Behavioral Sciences, Harvard T.H. Chan School of Public Health, 677 Huntington Ave, Boston, MA, United States; Department of Epidemiology, Boston University School of Public Health, 715 Albany st, Boston, MA, United States; Medical Scientist Training Program, School of Medicine, Virginia Commonwealth University, 1201 E Marshall St, Richmond, VA 23298, United States; Department of Social Behavioral Sciences, Harvard T.H. Chan School of Public Health, 677 Huntington Ave, Boston, MA, United States; Department of Social Behavioral Sciences, Harvard T.H. Chan School of Public Health, 677 Huntington Ave, Boston, MA, United States; Department of Primary Care and Medical Education, Institute of Medicine, University of Tsukuba, 1-1-1 Tennoudai, Tsukuba, Ibaraki 305-8575, Japan; Division of Public Health, Kitasato University School of Medicine, 1-15-1 Kitazato, Minami, Sagamihara, Kanagawa 252-0374, Japan; Tokyo Dental and Medical University, 1-5-45, Yushima, Bunkyo-ku, Tokyo, Japan; Chiba University, 1-33 Yayoicho, Inage Ward, Chiba, Japan; Department of Social Behavioral Sciences, Harvard T.H. Chan School of Public Health, 677 Huntington Ave, Boston, MA, United States; Department of Epidemiology, Harvard T.H. Chan School of Public Health, 677 Huntington Ave, Boston, MA, United States; Department of Social Behavioral Sciences, Harvard T.H. Chan School of Public Health, 677 Huntington Ave, Boston, MA, United States

**Keywords:** PTSD, depression, disaster, trauma, cognition, mediation

## Abstract

Emerging evidence links disaster-related home loss to increased cognitive impairment, yet the mechanisms underlying this association remain unclear. This study examines whether psychopathology and diminished social connection mediate the relationship between home loss and cognitive disability among older disaster survivors. We conducted a prospective cohort study of survivors of the 2011 Great East Japan Earthquake and Tsunami. Causal mediation analysis was used to estimate the role of post-disaster depressive symptoms, posttraumatic stress symptoms, social support, social participation, and social cohesion in mediating the association. Analyses adjusted for pre-disaster confounders. Among 3138 survivors, 140 experienced disaster-related home loss, and 498 experienced mild to severe cognitive disability. Home loss was associated with increased cognitive impairment, increased psychopathology, as well as decreased social connections. Forty-eight percent of the total effect of home loss on cognitive impairment was mediated by depressive symptoms, but not posttraumatic stress symptoms. Although the effect was marginal, 19% was also explained by a decline in social cohesion. Disaster-related home loss was associated with subsequent cognitive impairment through post-disaster depressive symptoms and decline in social cohesion. Group relocation and early intervention for those with depressive symptoms might ameliorate the adverse effects of home loss in cognitive disability.

## Introduction

Exposure to disaster-related trauma is a risk factor for cognitive impairment. A follow-up study of survivors of Hurricanes Katrina and Rita reported declines in short-term working memory performance among those who experienced disaster-related trauma, although longer-term associations with changes in cognitive ability were less evident.^1^^,^^2^

Potential mechanisms explaining the link between disaster-related trauma and cognitive impairment include changes in social connection, social participation, or community social cohesion triggered by disaster. For example, a follow-up study of older survivors of the 2011 Great East Japan Earthquake & Tsunami found that involuntary housing relocation was associated with diminished social support, social participation, and social cohesion, which are established risk factors for cognitive disability.^3^

Another mechanism linking home loss to cognitive disability is that disaster-related trauma increases the risks of psychopathology such as PTSD and depression, which are also known to adversely affect cognitive function.^4^^,^^5^ For example, studies among male US war veterans suggested that having PTSD after combat experience increased the risk of developing dementia.^6^^-^^8^ In a cohort of US women, a diagnosis of PTSD predicted subsequent declines in cognitive functioning, such as learning and recall,^9^^,^^10^ and this relationship was exacerbated by depressive symptoms.^9^^,^^11^ However, previous studies have not pinpointed the cause of PTSD, and it remains unclear whether posttraumatic symptoms caused by disaster-related trauma increase the risk of cognitive disability. Similarly, many study assessed PTSD symptoms retrospectively, leaving the temporal order unclear. Further, most studies were conducted among veteran or patient populations, and trauma exposure was not random, leaving concerns about the impact of selection bias.^6^^-^^10^ Although several meta-analyses have suggested an association between depression,^12^^-^^14^ trauma-related PTSD, and cognitive impairment, the conclusions are not consistent across these meta-analyses.^4^ Further, the Lancet Commission concluded that the evidence for the association is not established,^15^ as inconsistent findings between psychopathology and cognitive impairment stem from discrepancies in the methodologies, including data sources, follow-up periods, populations, and measurements of exposures and outcomes.

Few studies have adopted a causal mediation framework to examine how much of the effect of disaster-related trauma on cognitive disability can be explained. Understanding the underlying mechanisms could guide the development of post-disaster interventions to mitigate the adverse impact of trauma on the cognitive functions of older adult survivors. In this study, we sought to quantify whether specific pathways—onset of psychopathology and reduced social connectedness—underlie the association between disaster-related home loss and cognitive disability among older survivors of the 2011 Japanese earthquake and Tsunami using causal mediation analysis.^16^^,^^17^ Our analysis takes advantage of a unique prospective study, which was initiated before the disaster and followed by post-disaster surveys, providing rich pre-disaster information on participants without relying on their retrospective recall.^18^

## Methods

### Data

Our analysis is based on the Iwanuma Study. The city of Iwanuma is one of the field sites of the nationwide Japan Gerontological Evaluation Study (JAGES) cohort. The city was located approximately 80 km from the epicenter of the 2011 Great East Japan Earthquake and therefore directly affected by the tsunami, resulting in over 48% inundation of the land area.

The baseline survey of the Iwanuma Study was completed in August 2010, 7 months before the disaster. At baseline, a census was undertaken of all residents ≥65 years who were invited to participate in the study via mail (*n* = 8576). Of these, 4957 residents returned the mailed survey (response rate = 57.8%).^19^ The Great East Japan Earthquake occurred on March 11, 2011, and the earthquake and tsunami caused serious damage to Iwanuma City, killing 180 residents and damaging 5542 houses.^20^

**Figure 1:**
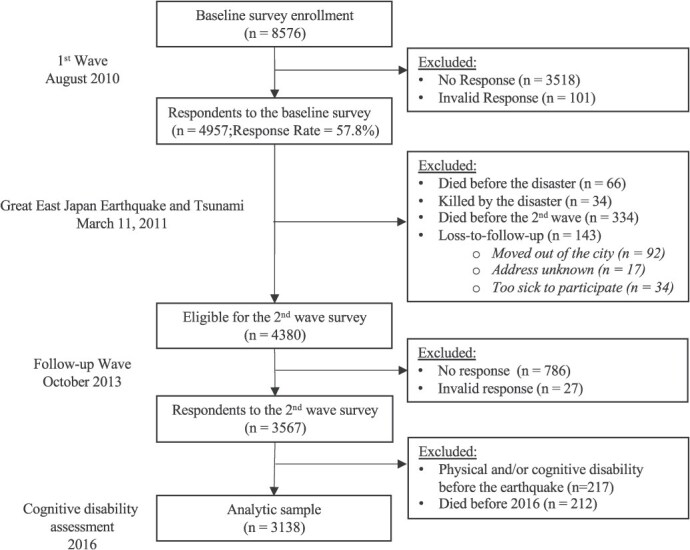
Sample flowchart.

In October 2013, approximately 2.5 years after the disaster, the study conducted a follow-up survey among survivors who responded to the baseline survey. In this post-disaster survey, participants retrospectively reported their disaster-related experiences, such as housing damage. Of the eligible survivors (*n* = 4380), responses were obtained from 3567 residents (follow-up rate = 81.4%). Participant records were then linked to the national Long Term Care Insurance (LTCI) registry which enabled us to identify individuals with preexisting physical or cognitive disability before the 2011 earthquake. We excluded individuals who had a physical and cognitive disability before the earthquake (*n* = 217). Further, we excluded those who died before 2016 (*n* = 212; ie, the time point for cognitive disability). Overall, our analytic sample was 3138 individuals (see Figure 1 for a flow chart).

### Measurement

#### Cognitive disability assessment

Our primary outcome was cognitive disability diagnosed in 2016, approximately 5.5 years after the disaster. Cognitive disability levels were assessed under Japan's national LTCI system with standardized in-home evaluation by trained investigators.^21^^,^^22^ Registration in the LTCI system is mandatory for everyone aged 40 years in Japan, and those who request long-term care services of any type (eg, in-home care services, daycare services) are assessed for eligibility, which is determined by cognitive disability levels or physical disability levels depending on the type of services individual requested.

Cognitive disability levels were assigned based on the individual's assessed levels of cognitive function, mental and behavioral symptoms, and ability to perform instrumental activities of daily living (Table S1). Based on these assessments, the applicant's cognitive functioning was classified into one of seven levels, with higher scores indicating greater cognitive disability (Table S2). The assessment of cognitive disability obtained from the LTCI registry has been shown to be strongly correlated with the Mini-Mental State Examination (Spearman's rank correlation ρ = −0.73, *P* < .001), the Clinical Dementia Rating scale (specificity and sensitivity, 0.88, *P* < .001), and physician diagnosis of dementia.^23^^,^^24^ We used the levels of cognitive disability for our outcome. Those who did not request long-term care health services were not evaluated for their cognitive status; thus, we assumed those individuals did not have any disabilities in our study.

#### Home loss

Our exposure, disaster-related home loss, was retrospectively reported in the 2013 follow-up survey. Respondents reported the degree of housing damage based on the result of the objective assessment by government property inspectors who classified the extent of housing damage into five levels (no damage, partial, minor, major, and complete destruction).^25^ The criteria for each level of housing damage are available in Table S3. We created a binary variable indicating complete destruction versus all other categories as follows the previous research examining the association between home loss and cognitive disability.^19^^,^^26^

#### Mediators

We assessed five potential mediators of the association between home loss and post-disaster cognitive disability during the post-disaster survey wave in 2013, including post-traumatic stress symptoms (PTSS), depressive symptoms, social support, informal socializing, and social cohesion. Of these, we assessed the same variables using the same items before the disaster, allowing us to assess the impact of changes.

Participants responded to the Screening Questionnaire for Disaster Mental Health (SQD), to assess PTSS among older adults.^27^ The SQD was originally developed based on the Post-Traumatic Symptom Scale (PTSS-10) and DSM-IV's diagnostic criteria for PTSD and Major Depressive Episode (MDE) to assess PTSD/PTSS among older adults affected by the Hanshin-Awaji Earthquake in Japan in 1995.^27^ The scale has been validated against the Clinician-Administered PTSD Scale and the Japanese-language version of the Clinician-Administered PTSD scale, and the internal consistency reliability (Cronbach's alpha = 0.77) has been tested. Among 15 items in the SQD, we identified PTSS using nine binary (yes/no) items related to symptoms like trouble sleeping, irritability, avoidance, and intrusive thoughts about the earthquake, following the instructions.^27^ We summed these responses to create a composite score (range, 0 [no PTSS] to 9 [highest PTSS]).

Depressive symptoms were assessed using the Japanese short version of the Geriatric Depression Scale-15 (GDS-15).^28^^,^^29^ The GDS-15 contains 15 items and has been validated with the Diagnostic and Statistical Manual of Mental Disorders, Fourth Edition, Text Revision (DSM-IV-TR) as a screening instrument for major depressive symptoms (sensitivity = 0.97, specificity = 0.95, Cronbach's α = 0.80).^29^ Participants were asked to respond to all 15 items with binary responses (yes/no). We reverse-coded five items and then summed them to create a composite score ranging from 0 to 15 (higher scores indicating more depressive symptoms).

For social support, respondents were asked to report whether they were receiving emotional and instrumental support from kin (eg, spouse, children living together, children living separately/relatives) and non-kin (eg, neighbors, friends, and others) after the disaster in 2013. In each instance, the question was asked, “Do you have anyone who you can talk to when you have concerns or complaints?” for emotional support and “Do you have anyone who can take care of you when you are sick for a few days?” for instrumental support. Respondents were asked to respond either yes or no to these questions, and we created four indicators of social support by adding up items based on the source and types, ie, 3 items for each (kin-emotional support, kin-instrumental support, non-kin emotional support, and non-kin instrumental support).

Social cohesion^30^ was assessed by three items, including residents' perceptions of trust in the community, levels of mutual help in the community, and community attachment. Each item was measured on a five-point scale ranging from 1 (not at all) to 5 (very much). We standardized each item, summed up all the items, and divided them by three to obtain a social cohesion indicator (Cronbach alpha = 0.75).

Informal socializing with neighbors was used as our measure of social participation. A short scale was constructed based on the frequency of meeting with friends (ie, how often do you see your friends?; 1: rarely to 6: almost every day), the number of friends met during the past month (ie, how many friends/acquaintances have you seen over the past month? 1, none to 5, 10 or more), the frequency of participation in a sports club (ie, how often do you attend activities in a sports club? 1, none to 6, almost every day), and the frequency of participation in hobby groups (ie, how often do you attend activities in hobby groups? 1, none to 6, almost every day). We standardized each item, summed all the items, and divided by four to obtain an informal socializing indicator (Cronbach alpha = 0.75).

### Covariates

We adjusted for baseline sociodemographic characteristics assessed in 2010, including continuous age, gender (men, women), marital status (married, not married), years of schooling (<9, 10-12, 13 years or longer, other), self-rated health (bad/not good, very good/good), the number of chronic conditions being treated (ranging from 0 to 20), continuous instrumental activity of daily living, continuous equivalized household income, and pre-disaster values of all the mediator variables, except for PTSS as it was not available in the pre-disaster wave. All mediators were treated as continuous variables.

### Statistical analysis

First, we examined (1) the associations between home loss and post-disaster levels of the mediator variables in 2013 (ie, the exposure–mediator associations represented by arrow A in Figure S1), (2) the association between home loss and cognitive disability level in 2016 (ie, the exposure–outcome association represented by the sum of the path A➔B and the arrow C), and (3) the associations between the post-disaster levels of the mediator variables in 2013 and cognitive disability level in 2016 (ie, the mediator–outcome associations represented by the arrow B) (Figure S1). We tested exposure–mediator interactions by adding an interaction term to the model. We used linear regression for all the analyses (ie, analyses [1], [2], and [3]). In analysis (3), we further adjusted for home loss status as it is a confounder of the mediator–outcome association.

Second, we performed causal mediation analysis using the R package “CMAverse.”^17^^,^^31^ We decomposed the total effect of an exposure into natural direct effect (NDE) and natural indirect effect (NIE). The NDE is defined as the effect of an exposure had a mediator been set to the value that would have been observed in the absence of the exposure, and the NIE is computed by subtracting the NDE from the total effect, representing the magnitude of the effect of the exposure through the mediator.^17^ The NDE can also be conceptualized as a weighted average of controlled direct effects (CDE) (the effect of an exposure while fixing the value of a mediator) across levels of a mediator. In the presence of an exposure–mediator interaction (ie, CDE varies by levels of a mediator), NDE and CDE will not be identical as NDE will be the weighted average of CDE. After estimating NDE and NIE, we calculated the proportion mediated (ie, a percentage of NIE compared to the total effect).

We performed a sensitivity analysis using a clinical cut-off for PTSD, as a previous study noted that the severity of PTSD matters in the association between PTSD and cognition.

We imputed missing data by chained equations and combined results from the five imputed datasets using the R package “MICE.”^32^ Standard errors were computed via bootstrapping with 1000 resampling.^33^

## Results

Table 1 summarizes the pre-disaster characteristics of the analytic sample according to home loss status. Among 3138 individuals, 140 people (4.5%) experienced home loss. In the overall sample, 74% were married, and 21% had an education attainment of 13 years or higher. Pre-disaster characteristics of participants were slightly different between those who experienced home loss and those who did not. For instance, of those who experienced home loss, only 8 individuals had 13 years or longer education (6.2%), compared to 22% of those who did not experience home loss. IADL score was slightly lower for those who experienced home loss.

**Table 1:** Pre-disaster characteristics of analytic sample by home loss status (*n* = 3138).

	**Overall**	**By home loss status** ^**a**^	
		**No home loss**	**Homeloss**
	***N* = 3057** ^**1**^	***N* = 2.917** ^**1**^	***N* = 140** ^**1**^
Age (years), mean (SD)	72.9 (5.8)	72.8 (5.8)	73.1 (6.1)
Gender, *n* (%)			
Men	1349 (44)	1291 (44)	58 (41)
Women	1708 (56)	1626 (56)	82 (59)
Marital status, *n* (%)			
Married	2188 (74)	2093 (74)	95 (76)
Not Married	771 (26)	741 (26)	30 (24)
Missing	98	83	15
Education, *n* (%)			
≤ 9 years	1004 (34)	918 (32)	86 (66)
10–12 years	1304 (44)	1269 (45)	35 (27)
13 years or longer	642 (22)	634 (22)	8 (6.2)
Other	23 (0.8)	22 (0.8)	1 (0.8)
Missing	84	74	10
Household income [10 000 yen], mean (SD)^b^	232 (141)	235 (141)	173 (128)
Missing	526	496	30
Self-rated Health, *n* (%)			
Bad/not good	463 (15)	441 (15)	22 (16)
Very good/good	2538 (85)	2426 (85)	112 (84)
Missing	56	50	6
Instrumental activities of daily lives, mean (SD)^c^	11.96 (1.68)	11.98 (1.64)	11.53 (2.29)
Missing	174	165	9
# of major diseases being treated, mean (SD)^d^	2.04 (1.29)	2.04 (1.29)	2.00 (1.24)
Missing	763	728	35
Depressive symptoms, mean (SD)^e^	3.4 (3.2)	3.4 (3.2)	3.8 (3.0)
Missing	394	369	25
Kin emotional support, mean (SD)	1.11 (0.76)	1.12 (0.76)	1.08 (0.86)
Missing	106	98	8
Non-kin emotional support, mean (SD)	0.56 (0.68)	0.56 (0.67)	0.64 (0.76)
Missing	106	98	8
Kin instrumental support, mean (SD)	1.33 (0.67)	1.33 (0.66)	1.37 (0.70)
Missing	68	62	6
Non-kin instrumental support, mean (SD)	0.11 (0.37)	0.11 (0.37)	0.09 (0.29)
Missing	68	62	6
Social cohesion, mean (SD)	0.00 (0.82)	0.00 (0.82)	0.00 (0.96)
Missing	134	122	12
Informal socializing, mean (SD)	−0.03 (0.76)	−0.02 (0.77)	−0.11 (0.70)
Missing	578	532	46

a The sample sizes for the subgroups do not add up to the size of the total samples (*n* = 3138) due to missing in the home loss status (*n* = 81). Missing home loss status was imputed in the subsequent analyses.

b Annual household income was divided by the square root of the number of household members to account for household size.

c IADL was measured by the 13-item Tokyo Metropolitan Institute of Gerontology Index of Competence. Scores ranged from 0 to 13 points for total IADL, 0-5 points for instrumental IADL, 0-4 points for intellectual IADL, and 0-4 points for social IADL, where smaller scores indicate lower functional independence.

d We calculated counts of current treatment for major diseases, including cancer, heart diseases, stroke, hypertension, diabetes, obesity, hyperlipidemia, osteoporosis, arthritis, fracture, respiratory diseases, gastrointestinal diseases, liver diseases, psychiatric diseases, dysphagia, visual impairment, hearing loss, dysuria, and insomnia.

e We used the Geriatric Depression Scale (range, 0-15 points; higher scores indicate more depressive symptoms) to assess depressive symptoms. It was used as continuous indicator.

**Figure 2:**
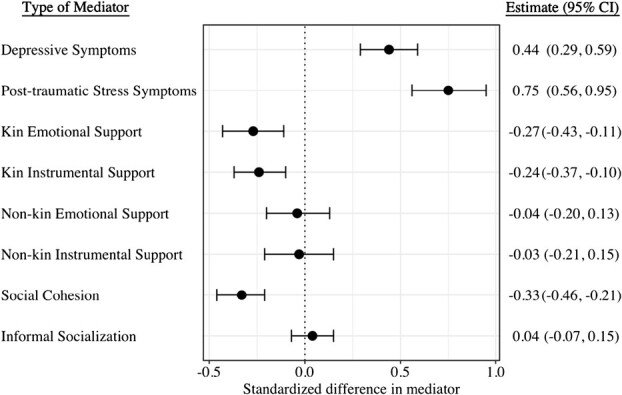
Associations between disaster-related home loss and potential mediators in 2013. Linear regression models were adjusted for pre-disaster characteristics including age, gender, marital status, education, household income, self-rated health, instrumental activities of daily living, the number of major diseases being treated, as well as pre-disaster values of all the mediator variables except for posttraumatic stress symptoms, which was not measured in the pre-disaster wave in 2010. Mediator variables were standardized (mean = 0 and standard deviation = 1) so that the effect estimates can be interpreted as standard deviation changes.

Figure 2 shows the association between home loss and potential mediators in 2013. Home loss was associated with significantly higher depressive symptoms scores (β, 0.44; 95% CI, 0.29-0.59) and posttraumatic stress symptoms scores (β, 0.75; 95% CI, 0.56-0.95). Home loss was also strongly associated with lower kin emotional support (β, −0.27; 95% CI, −0.43 to −0.11), kin instrumental support (β, −0.24; 95% CI, −0.37 to −0.10), and social cohesion (β, −0.33; 95% CI, −0.46 to −0.21). There was no strong association between home loss and non-kin emotional support (β, −0.04; 95% CI, −0.20 to 0.13), non-kin instrumental support (β, −0.03; 95% CI, −0.21 to 0.15), and informal socializing (β, 0.04; 95% CI, −0.07 to 0.15).

**Figure 3:**
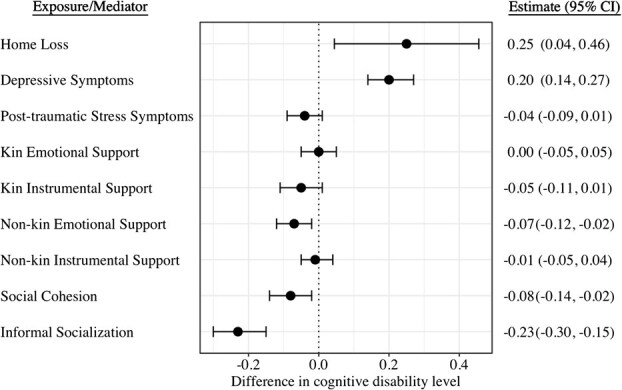
Associations between home loss/mediators and cognitive disability levels in 2016. Home loss was binary. Mediators were reported in 2013 and standardized so that the estimates represent differences in the cognitive disability level per one SD change in the mediator values. Linear regression models were adjusted for pre-disaster characteristics including age, gender, marital status, education, household income, self-rated health, instrumental activities of daily living, the number of major diseases being treated, as well as pre-disaster values of all the mediator variables except for posttraumatic stress symptoms, which was not measured in the pre-disaster wave in 2010. Models for the mediators were further adjusted for home loss status. Levels of certified cognitive disability ranged from 0 (no cognitive deficits) to 7 (needs constant treatment in a specialized medical facility) according to the severity of their cognitive disability. Thus, positive values for effect estimates indicate greater levels of cognitive disability.

Figure 3 shows the associations between home loss or mediators and cognitive disability levels in 2016. We found strong evidence of association for home loss (β, 0.25; 95% CI, 0.04-0.46), depressive symptoms (β, 0.20; 95% CI, 0.14-0.27), non-kin emotional support (β, −0.07; 95% CI, −0.12 to −0.02), social cohesion (β, −0.08; 95% CI, −0.14 to −0.02), and informal socializing (β, −0.23; 95% CI, −0.30 to −0.15). For other mediators, there was no strong evidence of association with cognitive disability levels. We also found no strong evidence of exposure–mediator interactions (Table S4; eg, an interaction between home loss exposure and depressive symptoms as a mediator: β = 0.07; 95% CI, −0.15 to 0.29; *P* = .520). Table 2 shows the results of the causal effect decomposition of the total effect of home loss on cognitive disability levels mediated through post-disaster social support, social cohesion, informal socializing, depressive symptoms, and PTSS. We examined only one potential mediator at a time to examine the impact of each mediator separately. For the total effect of home loss on cognitive disability levels, we observed an indirect effect mediated through post-disaster depressive symptoms (β, 0.14; 95% CI, 0.05-0.26). Although the effect was not significant, we observed a weak association for social cohesion (β, 0.06; 95% CI, 0.00-0.14). The proportion mediated was 47.8% (*P* ≤ .001) for post-disaster depressive symptoms and 19.4% for post-disaster social cohesion (*P* = .08). There was no strong evidence of indirect effect through other mediators, including PTSS, social support, or informal socializing.

**Table 2:** Decomposition of the estimated total effect of disaster-related home loss on cognitively disability levels in 2016.

**Mediators in 2013**	**Total effect of home loss**		**Natural indirect effect**		**Natural direct effect**		**Proportion mediated**
	**Estimate (95% CI)**	***P***	**Estimate (95% CI)**	***P***	**Estimate (95% CI)**	***P***	**%**
Depressive symptoms	0.29 (0.02-0.50)	.04	0.14 (0.05-0.26)	<.001	0.15 (−0.12 to 0.41)	032	47.8
Posttraumatic stress symptoms	0.32 (0.07-0.64)	<.001	−0.10 (−0.26 to 0.08)	.36	0.42 (0.06-0.87)	<.001	−31.4
Kin emotional support	0.30 (0.04-0.55)	<.001	0.03 (−0.01 to 0.10)	.16	0.27 (0.03-0.51)	.04	11.2
Kin instrumental support	0.30 (0.03-0.44)	<.001	0.00 (−0.05 to 0.03)	.76	0.30 (0.03-0.45)	.04	−0.59
Non-kin emotional support	0.28 (0.06-0.53)	.04	0.02 (−0.02 to 0.08)	.40	0.27 (0.02-0.53)	.04	6.01
Non-kin instrumental support	0.28 (0.08-0.51)	<.001	0.00 (−0.01 to 0.02)	.68	0.28 (0.08-0.51)	<.001	−0.46
Social cohesion	0.31 (0.15-0.50)	<.001	0.06 (0.00-0.14)	.08	0.25 (0.03-0.42)	<.001	19.4
Informal Socializing	0.29 (0.02-0.58)	.04	0.00 (−0.02 to 0.01)	.72	0.29 (0.02-0.59)	.04	−0.28

We decomposed the total effect of disaster-related home loss on cognitive disability levels in 2016 using regression-based causal mediation analysis. For each candidate mediator, we conducted causal mediation analysis that specifies two models: one for the mediator and one for the outcome with exposure–mediator interaction. Both models were adjusted for pre-disaster characteristics including age, gender, marital status, education, household income, self-rated health, instrumental activities of daily living, the number of major diseases being treated, as well as pre-disaster values of all the mediator variables except for posttraumatic stress symptoms, which was not measured in the pre-disaster wave in 2010. We computed 95% CIs and *P* values based on standard errors obtained via bootstrapping with 100 resampling. Levels of certified cognitive disability ranged from 0 (no cognitive deficits) to 7 (needs constant treatment in a specialized medical facility) according to the severity of their cognitive disability. Thus, positive values for effect estimates indicate greater levels of cognitive disability. Proportion mediated was computed by dividing a natural indirect effect by a total effect.

We conducted a sensitivity analysis using a clinical cutoff of PTSD, as the previous study indicates that severity affects the results. Table S5 shows that the trend was similar to our main analysis, that PTSS did not explain the total effect of home loss and cognitive disabilities.

## Discussion

To our knowledge, this is the first causal mediation analysis examining the potential pathways linking disaster-related home loss to the onset of post-disaster cognitive disability. Three notable findings emerged. First, disaster-related home loss was associated with an increased risk of depressive symptoms, PTSS, lower social cohesion, kin-emotional support, and kin-instrumental support, while we found no association for non-kin emotional/instrumental support and informal socializing. Second, we found that depressive symptoms and social cohesion mediated the association between home loss and cognitive disability. Specifically, 48% of the association between disaster-related home loss and cognitive disability was mediated through depressive symptoms, while 19% was mediated through decline in social cohesion. Lastly, we found no evidence of mediation by other variables, including PTSS.

Our findings on the association between depressive symptoms and cognitive disability align with existing literature.^15^ Although this association is typically considered bidirectional, our study design allowed us to control for pre-disaster depression status, strengthening the case that depression resulting from disaster-related home loss contributes to subsequent cognitive impairment.^14^

Several potential mechanisms may explain how depression leads to cognitive disability in disaster survivors. First, depression might impair self-care behaviors and reduce social connectedness, which is particularly detrimental for older survivors forced to relocate to new environments. Second, biological pathways may play a role, including hyperactivation of the hypothalamic–pituitary–adrenal (HPA) axis, and impaired brain glucose metabolism—all of which can contribute to neurodegeneration following stress exposure and depression.^34^^,^^35^ Additionally, depression may trigger proinflammatory responses in the brain that accelerate the degenerative changes characteristic of dementia.^35^

By contrast, we found that PTSS was not associated with cognitive impairment nor mediated the association between disaster-related home loss and cognitive impairment, which is somewhat inconsistent with existing evidence.^12^^,^^34^ For instance, existing meta-analyses suggest that PTSD is likely to cause cognitive impairment or dementia, with HRs ranging from 1.70 to 4.37 between 1996 and 2021.^14^^,^^15^ These studies also pointed out that observed PTSD-cognitive associations are higher among people with comorbid depression. However, our results still showed no association between PTSD and cognition after removing pre-disaster depression status. While these meta-analyses overall support an association between PTSD and cognitive impairment, methodological heterogeneity has also been noted across studies.^14^^,^^15^ Studies with shorter follow-up periods tend not to observe cognitive impairment following PTSD,^36^ while other studies reported that PTSD affects only specific dimensions of cognitive function, including learning or recall.^10^ The follow-up period of our study was 5.5 years, which is shorter than other studies that reported an association between PTSD and dementia. Similarly, our outcome measure is based on a global rating of dementia symptoms and clinically manifest cognitive disability and did not assess cognitive function in specific domains. It is possible that PTSD is associated with specific dimensions of cognitive impairment, such as learning or recall,^10^ making it harder to detect an association when using global measures of cognitive disability.

The discrepancy between previous studies and our study may suggest different mechanisms related to the pathways to cognitive disability. Our findings highlight that the association between PTSS/PTSD and cognitive impairment is heterogeneous and not as prominent as depression.^12^^,^^37^ Further research is needed to consider whether specific types of traumatic experiences differ in their association with PTSD, as well as whether the severity and duration of PTSD/PTSS matter.

We found that both post-disaster social cohesion and informal socializing were linked to later cognitive disability, supporting previous studies on their protective role against cognitive disability after disaster trauma.^30^^,^^38^ Interestingly, we have previously reported that among individuals who lost their homes, group relocation to temporary housing (ie, moving people together from the same neighborhoods) preserved social cohesion and informal socializing. In contrast, survivors who were individually relocated into temporary housing experienced a decrease in informal socializing and social cohesion. We observed strong evidence of an association between informal socialization and cognitive disability, but did not find evidence of an association between home loss and informal socialization. This finding aligns with our previous research showing that the type of relocation, rather than home loss itself, affects social connections.^3^ Consequently, this lack of association between home loss and informal socialization likely explains why we found no evidence for the mediating effect of informal socialization.

We also found that those who experienced home loss reported lower kin emotional and instrumental support compared to those who escaped home loss. It is possible that home loss was correlated with the loss of relatives or friends, which may have resulted in a decrease in kin emotional/instrumental support. While we found differences in kin social support by home loss status, we did not find any difference in non-kin social support. Any of the social support-related exposure was associated with cognitive disability.

Our study has four limitations. First, selection bias due to sample attrition is possible. When we compared participants included versus excluded in the analysis, there were differences in their pre-disaster characteristics. For example, those who were excluded from the analysis were likely to be older, non-married, report worse self-rated health, or worse depressive symptoms compared to those who were included (Table S6). Such attrition may have resulted in the underestimation of the exposure–outcome associations.^39^ Second, the identification assumptions for the causal mediation analysis may not be met. Causal mediation analysis requires: (1) no unmeasured exposure–outcome confounding, (2) no unmeasured mediator–outcome confounding, (3) no unmeasured exposure–mediator confounding, and (4) no mediator–outcome confounding affected by the exposure. Although we adjusted for a rich set of preexposure confounders including pre-disaster household income, bias may remain due to unmeasured confounding (eg, pre-disaster home value). Third, we examined a single mediator at a time rather than simultaneously modeling multiple mediators. As the mediators are likely correlated with each other, the estimated NIEs capture some overlapping pathways. Fourth, although the relationship between disaster experiences, mediators, and subsequent cognitive abilities may vary according to individual characteristics (such as socioeconomic status or gender),^40^ we estimated average effects. Fifth, our follow-up period may be too short to detect the mediating effect of PTSD in the association between disaster-related home loss and cognitive disability. As several studies have pointed out that a longer duration of follow-up predicts PTSD-cognitive associations,^15^ further research with longer follow-up data is warranted. Sixth, although registration in the LTCI system is mandatory for everyone aged 40 years or older in Japan, those who request long-term care services of any type (eg, in-home care services, daycare services) are assessed for eligibility by undergoing a cognitive and physical disability assessment. This assessment procedure may overlook those with very mild conditions who do not need any support. This measurement error may cause an underestimation of our results; however, if the error is non-differential, our direct and indirect effects may be unbiased. Lastly, our results may have suffered from limited power due to sample size and the statistical analysis we employed. Further study with a larger sample size to confirm our results is needed.

In summary, the association of disaster-related home loss with subsequent cognitive disability appeared to be partially mediated through post-disaster depressive symptoms and loss of social cohesion. Group relocation to preserve social ties and early intervention for those with depressive symptoms may protect the cognitive health of older disaster survivors.

## Supplementary Material

Web_Material_kwaf208

## Data Availability

The data set is available upon request.
