# Supplementary material for: Disaster-related home loss, mental health, and risk of cognitive disability: causal mediation analysis using longitudinal data of disaster survivors
Source: Am J Epidemiol. 2025 Sep 22;194(12):3501–9. doi: 10.1093/aje/kwaf208 (PMC12671964; doi:10.1093/aje/kwaf208)
Supplement: Web_Material_kwaf208 [file web_material_kwaf208.zip › Supplementary_Jul25.docx]

Supplementary materials

**Title:** Disaster-related Home Loss, Mental Health, and Risk of Cognitive Disability: Causal Mediation Analysis Using Longitudinal Data of Disaster Survivors

**Authors:** **:** Sakurako S.Okuzono1*, Koichiro Shiba2, David T. Zhu3, Sarah Oh1, Yu-Tien Hsu1, Aki Yazawa4, Hiroyuki Hikichi5, Jun Aida6, Katsunori Kondo7, Henning Tiemeier1,8, Ichiro Kawachi1

**Table S1. Questionnaires to Assess the Level of Cognitive Function, Mental and Behavioral Disorders, an Activity of Daily Living and Instrumental Activities of Daily Living used in the Japan’s Long-Term Insurance System (Translated into English)**

**Table S2. Levels of Cognitive Disability Levels and Criterion**

**Table S3. Levels of Housing Damage and Criterion Certifying by Local Governments**

**Table S4. Results of testing exposure-mediator interaction.**

**Table S5. Decomposition of the estimated total effect of disaster-related home loss on cognitive disability levels in 2016 by PTSS**

**Table S6. Pre-disaster characteristics of whole samples stratified by analyzed status.**

**Figure S1. Causal diagram illustrating hypothesized relationships between the key variables.**

**Table S1. Questionnaires to Assess the Level of Cognitive Function, Mental and Behavioral Disorders, an Activity of Daily Living and Instrumental Activities of Daily Living used in the Japan’s Long-Term Insurance System (Translated into English)**

| 3-1 | Please circle one number that applies to applicant’s communication. | 1. Able to communicate with others 2. Sometimes 3. Not at all 4. No |
| --- | --- | --- |
| 3-2 | Please circle one number that applies to applicant’s understanding in daily routine | 1. Able to do it 2. Unable to do it |
| 3-3 | Please circle one number that applies to applicant’s understanding in their birthday and name | 1. Able to do it 2. Unable to do it |
| 3-4 | Please circle one number that applies to applicant’s short-time memory | 1. Able to do it 2. Unable to do it |
| 3-5 | Please circle one number that applies to applicant’s ability to say their name | 1. Able to do it 2. Unable to do it |
| 3-6 | Please circle one number that applies to applicant’s understanding in current season | 1. Able to do it 2. Unable to do it |
| 3-7 | Please circle one number that applies to applicant’s understanding in the place | 1. Able to do it 2. Unable to do it |
| 3-8 | Please circle one number that applies to applicant’s regarding wandering | 1. Not at all 2. Sometimes 3. Often |
| 3-9 | Please circle one number that applies to applicant’s regarding not be able to come home by oneself | 1. Not at all 2. Sometimes 3. Often |
| 4-1 | Please circle only one number that applies to applicant’s victimization such as having things stolen | 1. Never 2. Sometimes 3. Yes |
| 4-2 | Please circle one item for each of the following that applies to applicant in terms of making up stories. | 1. Never 2. Sometimes 3. Yes |
| 4-3 | Please circle one number that applies to applicant’s emotional instability such as crying or laughing. | 1. Never 2. Sometimes 3. Yes |
| 4-4 | Please circle one item that applies to applicant’s experience of day/night reversal | 1. Never 2. Sometimes 3. Yes |
| 4-5 | Please circle one item for persistent talking about the same thing. | 1. Never 2. Sometimes 3. Yes |
| 4-6 | Please circle one item for loudness. | 1. Never 2. Sometimes 3. Yes |
| 4-7 | Please circle one item for each of the following that applies to applicant regarding resistance to nursing care. | 1. Never 2. Sometimes 3. Yes |
| 4-8 | Please circle one item that applies to applicant’s concern about the fact that the patient becomes restless, saying things like "I'm going home". | 1. Never 2. Sometimes 3. Yes |
| 4-9 | Please circle one item for each of the following that describes how applicant wants to go outside alone and caregiver cannot take their eyes off from them. | 1. Never 2. Sometimes 3. Yes |
| 4-10 | Please circle one item that applies to the applicants regarding collecting things | 1. Never 2. Sometimes 3. Yes |
| 4-11 | Please circle one item for breaking things or tearing clothes. | 1. Never 2. Sometimes 3. Yes |
| 4-12 | Please circle one item for severe memory loss. | 1. No 2. Sometimes 3. Yes |
| 4-13 | Please circle one item for talking or laughing to yourself for no reason. | 1. Never 2. Sometimes 3. Yes |
| 4-14 | Please circle one item for selfishness.  What’s this?? | 1. Never 2. Sometimes 3. Yes |
| 4-15 | Please circle one item for each of the following that applies to ability to keep a conversation going. | 1. No 2. Sometimes 3. Yes |
| 5-1 | Please circle one item for each of the following that applies to applicants regarding taking medication | 1. Independent 2. Partly support 3. Full support |
| 5-2 | Please circle one item for each of the following that applies to applicants’ money management | 1. Independent 2. Partly support 3. Full support |
| 5-3 | Please circle one item for each of the following that applies to applicants’ ability to make decision for daily living | 1. Able to do it 2. Able to do it except for special occasion 3. Unable to do it occasionally 4. Not at all |
| 5-4 | Please circle one item for each of the following that applies to applicants’ maladjustment to groups | 1. Not at all 2. Sometimes 3. Yes |
| 5-5 | Please circle one item for each of the following that applies to applicants’ regarding shopping ability | 1. Able to do it 2. Needs support 3. Needs support partly 4. Needs full support |
| 5-6 | Please circle one item for each of the following that applies to applicants’ regarding cooking | 1. Able to do it 2. Needs support 3. Needs support partly 4. Needs full support |

**Table S2. Levels of Cognitive Disability Levels and Criterion**

| **Rank** | **Levels** | **Criterion** | **Examples of symptoms or behaviors** |
| --- | --- | --- | --- |
| NA | 1 | No cognitive impairment. Completely independent. | NA |
| Ⅰ | 2 | Although some cognitive impairment, there is no barriers in daily living and socially independent. | NA |
| Ⅱ | There are some symptoms, behaviors, or communication difficulties that cause barriers in daily life. However, individuals can be independent if someone pays attention to them. | | |
| Ⅱa | 3 | Conditions of Ⅱ can be observed outside home. | Frequently gets lost, or makes mistakes in shopping, doing paperwork, money management, or other tasks that were previously easy to do. |
| Ⅱb | 4 | Conditions of Ⅱ can be observed at home, as well as outside home. | Unable to manage medication, unable to answer the phone or answer visitors. |
| Ⅲ | There are some symptoms, behaviors, or communication difficulties that cause barriers in daily life, and requiring nursing care. | | |
| Ⅲa | 5 | Condition of Ⅲ can be observed during the day. | Unable to change clothes, eat, defecate, urinate (or take a long time to perform these functions). Uncontrollable behaviors such as putting things in mouth, incontinence, risk of starting fires, lack of hygiene, sexually inappropriate behaviors. |
| Ⅲb | 6 | Condition of Ⅲ can be observed during the night. |  |
| Ⅳ | 7 | There are frequent symptoms, behaviors, or communication difficulties that cause barriers in daily life, and requiring nursing care all the time. |  |
| M | 8 | Significant psychiatric symptoms, problematic behavior, or serious physical illness requiring specialized medical care. | Delirium, delusion, agitation, psychiatric symptoms such as self-injury and harm, and problematic behaviors caused by psychiatric symptoms. |

**Table S3. Levels of Housing Damage and Criterion Certifying by Local Governments**

| **Grade** | **Criterion^a^** |
| --- | --- |
| No Damage | Not affected. |
| Partial | Under 20% structural damage or inundation below the floor. |
| Minor | 20% to 40 % structural damage or inundation above the floor. |
| Major | 40% to 50% structural damage or inundation approximately 1 meter above the floor. |
| Complete Destruction (Home Loss) | Over 50% structural damage, inundation up to ceiling in the first floor, or completely washed away. Uninhabitable beyond repair. |

^a^Structural damage was observed in roof, walls, and foundation

**Table S4. Results of testing exposure-mediator interaction.**

| Exposure*Mediator | Estimate (95% CI) | P-value |
| --- | --- | --- |
| Depressive symptoms*home loss | 0.07 (-0.15, 0.29) | 0.520 |
| Post-traumatic stress symptoms*home loss | -0.07 (-0.26, 0.11) | 0.460 |
| Kin emotional support*home loss | -0.14 (-0.36, 0.07) | 0.190 |
| Kin instrumental support*home loss | 0.06 (-0.13, 0.26) | 0.520 |
| Non-kin emotional support*home loss | -0.15 (-0.35, 0.05) | 0.140 |
| Non-kin instrumental support*home loss | 0.01 (-0.22, 0.25) | 0.900 |
| Social cohesion*home loss | -0.07 (-0.29, 0.14) | 0.520 |
| Informal Socializing*home loss | 0.21 (-0.09, 0.5) | 0.170 |

Home loss was binary. Mediators were reported in 2013 and standardized so that the estimates represent differences in the cognitive disability level per one SD change in the mediator values. Linear regression models were adjusted for pre-disaster characteristics including age, gender, marital status, education, household income, self-rated health, instrumental activities of daily living, the number of major diseases being treated, as well as pre-disaster values of all the mediator variables except for post-traumatic stress symptoms, which was not measured in the pre-disaster wave in 2010. Models for the mediators were further adjusted for home loss status. Levels of certified cognitive disability ranged from 0 (no cognitive deficits) to 7 (needs constant treatment in a specialized medical facility) according to the severity of their cognitive disability. Thus, positive values for effect estimates indicate greater levels of cognitive disability

**Table S5. Decomposition of the estimated total effect of disaster-related home loss on cognitive disability levels in 2016 by PTSS with clinical cut-off**

|  | Estimate | 95% CI | | P-value |
| --- | --- | --- | --- | --- |
|  |  | Lower | Upper |  |
| Natural direct effect | 0.28 | 0.02 | 0.52 | 0.040 |
| Natural indirect effect | 0.00 | -0.08 | 0.07 | 0.760 |
| Total effect of home loss | 0.28 | 0.05 | 0.46 | 0.000 |
| Proportion mediated | 0.00 | -0.33 | 0.91 | 0.760 |

We decomposed the total effect of disaster-related home loss on cognitive disability levels in 2016 using regression-based causal mediation analysis. For each candidate mediator, we conducted causal mediation analysis that specifies two models: one for the mediator and one for the outcome with exposure-mediator interaction. Both models were adjusted for pre-disaster characteristics including age, gender, marital status, education, household income, self-rated health, instrumental activities of daily living, the number of major diseases being treated, as well as pre-disaster values of all the mediator variables except for post-traumatic stress symptoms, which was not measured in the pre-disaster wave in 2010. We computed 95% confidence intervals and p-values based on standard errors obtained via bootstrapping with 100 resampling. Levels of certified cognitive disability ranged from 0 (no cognitive deficits) to 7 (needs constant treatment in a specialized medical facility) according to the severity of their cognitive disability. Thus, positive values for effect estimates indicate greater levels of cognitive disability. Proportion mediated was computed by dividing a natural indirect effect by a total effect

**Table S6. Pre-disaster characteristics of whole samples stratified by analyzed status.**

|  | **Overall** | **Status** | |
| --- | --- | --- | --- |
|  |  | **Excluded** | **Included** |
|  | N = 5,048 | N = 1,910 | N = 3,138 |
| **Home loss by disaster** |  |  |  |
| No | 3,307 (95%) | 390 (95%) | 2,917 (95%) |
| Yes | 159 (4.6%) | 19 (4.6%) | 140 (4.6%) |
| Missing | 1,582 | 1,501 | 81 |
| **Age (years), mean (SD)** | 75 (7) | 78 (8) | 73 (6) |
| **Gender, n (%)** |  |  |  |
| Men | 2,193 (43%) | 817 (43%) | 1,376 (44%) |
| Women | 2,855 (57%) | 1,093 (57%) | 1,762 (56%) |
| **Marital status, n (%)** |  |  |  |
| Married | 3,300 (68%) | 1,065 (59%) | 2,235 (74%) |
| Not Married | 1,529 (32%) | 731 (41%) | 798 (26%) |
| Missing | 219 | 114 | 105 |
| **Education, n (%)** |  |  |  |
| ≤9 years | 1,845 (38%) | 804 (45%) | 1,041 (34%) |
| 10-12 years | 1,996 (41%) | 660 (37%) | 1,336 (44%) |
| 13 years or longer | 945 (20%) | 295 (16%) | 650 (21%) |
| Other | 55 (1.1%) | 31 (1.7%) | 24 (0.8%) |
| Missing | 207 | 120 | 87 |
| **Household income [10,000 yen], mean (SD)**^a^ | 229 (148) | 224 (159) | 231 (141) |
| Missing | 1,027 | 475 | 552 |
| **Self-rated Health, n(%)** |  |  |  |
| Bad/not good | 1,169 (24%) | 692 (37%) | 477 (15%) |
| Very good/good | 3,788 (76%) | 1,187 (63%) | 2,601 (85%) |
| Missing | 91 | 31 | 60 |
| **Instrumental Activities of Daily Lives, mean (SD)**^b^ | 10.9 (3.2) | 9.2 (4.3) | 11.9 (1.7) |
| Missing | 377 | 197 | 180 |
| **# of major diseases being treated, mean (SD)**^c^ | 2.27 (1.52) | 2.58 (1.75) | 2.05 (1.30) |
| Missing | 1,115 | 332 | 783 |
| **Depressive symptoms, mean (SD)**^d^ | 4.0 (3.6) | 5.0 (4.0) | 3.4 (3.3) |
| Missing | 767 | 352 | 415 |
| **Kin emotional support, mean (SD)** | 1.06 (0.76) | 0.97 (0.75) | 1.11 (0.76) |
| Missing | 250 | 139 | 111 |
| **Non-kin emotional support, mean (SD)** | 0.51 (0.66) | 0.43 (0.62) | 0.56 (0.67) |
| Missing | 250 | 139 | 111 |
| **Kin instrumental support, mean (SD)** | 1.27 (0.68) | 1.17 (0.69) | 1.33 (0.66) |
| Missing | 176 | 102 | 74 |
| **Non-kin instrumental support, mean (SD)** | 0.12 (0.37) | 0.13 (0.38) | 0.11 (0.36) |
| Missing | 176 | 102 | 74 |
| **Social cohesion, mean (SD)** | 0.00 (0.83) | -0.08 (0.91) | 0.05 (0.78) |
| Missing | 306 | 164 | 142 |
| **Informal socializing, mean (SD)** | -0.03 (0.77) | -0.26 (0.75) | 0.09 (0.76) |
| Missing | 1,089 | 488 | 601 |

SD: standard deviation.

^a^ Annual household income was divided by the square root of the number of household members to account for household size.

^b^ IADL was measured by the 13-item Tokyo Metropolitan Institute of Gerontology Index of Competence. Scores ranged from 0-13 points for total IADL, 0-5 points for instrumental IADL, 0-4 points for intellectual IADL, and 0-4 points for social IADL, where smaller scores indicate lower functional independence.

^c^ We calculated counts of current treatment for major diseases, including cancer, heart diseases, stroke, hypertension, diabetes, obesity, hyperlipidemia, osteoporosis, arthritis, fracture, respiratory diseases, gastrointestinal diseases, liver diseases, psychiatric diseases, dysphagia, visual impairment, hearing loss, dysuria, and insomnia.

^d^ We used the Geriatric Depression Scale (range: 0-15 points; higher scores indicate more depressive symptoms) to assess depressive symptoms. We used a cut-off of ≥5 points to define moderate/severe depressive symptoms.

**Figure S1. Causal diagram illustrating hypothesized relationships between the key variables.**

In this study, we first examined (i) the associations between disaster-related home loss (i.e., happened in 201 but assessed in 2013) and post-disaster levels of the mediator variables in 2013 (i.e., the exposure-mediator associations represented by the arrow A). The mediator variables included depressive symptoms, post-traumatic stress symptoms, social support (kin emotional, kin instrumental, non-kin emotional, and non-kin instrumental support), social cohesion, and informal socializing. We also estimated (ii) the association between disaster-related home loss and cognitive disability level in 2016 (i.e., the exposure-outcome association represented by the sum of the path A🡪B and the arrow C) and (iii) the associations between the post-disaster levels of the mediator variables in 2013 and cognitive disability level in 2016 (i.e., the mediator-outcome associations represented by the arrow B). The subsequent formal causal mediation analysis decomposed the total effect of the disaster-related home loss on cognitive disability levels into natural indirect effect (the path A🡪B) and the direct effect (the arrow C).
